# Availability and Use of Digital Technology Among Women With Polycystic Ovary Syndrome: Scoping Review

**DOI:** 10.2196/68469

**Published:** 2025-06-12

**Authors:** Pamela J Wright, Charlotte Burts, Carolyn Harmon, Cynthia F Corbett

**Affiliations:** 1 University of South Carolina, 1601 Greene Street, Columbia, SC, 29208, United States, 1 803-777-6039

**Keywords:** polycystic ovary syndrome, digital technology, mobile apps, digital health, self-management, mobile phone

## Abstract

**Background:**

Polycystic ovary syndrome (PCOS) is a common endocrinopathy among women that requires self-management to improve mental and physical health outcomes and reduce risk of comorbidity. Digital technology has rapidly emerged as a valuable self-management tool for people with chronic health conditions. However, little is known about the digital technology available for and used by women with PCOS.

**Objective:**

The purpose of this scoping review was to identify what is known about digital technology currently available and used by women with PCOS for PCOS-specific knowledge, self-management, or social support.

**Methods:**

The databases PubMed, Embase, CINAHL, and Compendex were searched using Medical Subject Headings terms for PCOS, digital technology, health knowledge, self-management, and social support. Inclusion criteria were full-text, peer-reviewed publications of primary research from 2010 to 2025 in English about digital technology used for PCOS-specific knowledge, self-management, or social support by women aged 18 years and older with PCOS. Exclusion criteria were articles about pediatric populations and digital technology used for intervention recruitment or by health care providers to diagnose or treat patients.

**Results:**

In total, 34 full-text articles met the inclusion criteria. Given the scope of digital technology, eligible studies were grouped into 7 domains: mobile apps (n=14), internet-based programs (eg, Google; n=6), social media (n=6), SMS text message (n=2), machine learning (n=2), artificial intelligence (eg, ChatGPT [OpenAI]; n=3), and web-based intervention platforms (n=1). Findings highlighted participants’ varied perceptions of technology usefulness based on reliability of health care information, application features, accuracy of PCOS or fertility prediction, social group engagement, user-friendly interfaces, cultural sensitivity, and accessibility.

**Conclusions:**

There is potential for digital technology to transform PCOS self-management, but further design and development are needed to optimize the technologies for women with PCOS. Future research should focus on including end users during the design phase of digital technology, refining predictive models, improving app inclusivity, conducting frequent reliability testing, and enhancing user engagement and support via additional features to promote more comprehensive self-management of PCOS.

## Introduction

Polycystic ovary syndrome (PCOS) is a complex, heterogenous collection of symptoms due to hormonal dysregulation [1], and it is the most common endocrinopathy among women across all races and ethnicities [2]. Characterized by hyperandrogenism, ovulatory dysfunction, or polycystic ovaries, PCOS often manifests with a range of symptoms such as irregular menstrual cycles, hirsutism, and metabolic disturbances [1]. Obesity, insulin resistance, and dyslipidemia are common clinical features in women with PCOS and increase the risk for cardiometabolic diseases and reproductive cancers by ≥50% [3] while negatively impacting women’s health-related quality-of-life and psychological morbidity [4]. Treatment guidelines for PCOS are complex and multifaceted, as the Endocrine Society Practice Guidelines recommend medical adherence, cognitive strategies, and lifestyle changes to self-manage day-to-day PCOS symptoms [5] and reduce affected women’s risk of comorbid conditions (eg, cardiometabolic) [6]. Thus, there is an urgent need for innovative approaches to help women with PCOS effectively manage their condition.

Digital technology is defined as tools, systems, or devices that can generate, store, or process data, such as smartphone apps, wearable trackers, online platforms, and social media communities [7]. Over the past 3 decades, a range of digital technologies to support health have rapidly developed and realized widespread adoption. Studies have revealed that women are more frequent users of online health information than men [8]. However, the gendered dimensions of using digital technologies to self-manage chronic health conditions has received little attention. Despite the prevalence of chronic health conditions among women across the lifespan, the little research available is primarily about pregnancy-related conditions or the perinatal period [9,10].

As a chronic condition, PCOS transcends the reproductive years. Study findings indicate that women with PCOS report inadequate information and disconcerting interactions with health care professionals [11,12], such that many women with PCOS pursue health care information and social support via the internet [13,14]. Approximately 98% of women with PCOS searched for PCOS-specific information on Google and 19% joined PCOS support groups or forums found during web-based searches [10]. However, little is known about the breadth of digital technology available for or used by women with PCOS or the features offered to help promote health for women with PCOS. Thus, the purpose of this scoping review was to identify available digital technology used by women with PCOS for PCOS-specific knowledge, self-management, or social support.

## Methods

### Overview

A scoping review was chosen due to the broad nature of the research question in a relatively new area of interest, that being both digital technology and its purposeful use among women with PCOS. A scoping review is ideal when attempting to categorize the volume, nature, and features of emerging research [15]. This scoping review was conducted using the updated Joanna Briggs Institute (JBI) guidance for scoping reviews [16].

The research question guiding this review was: “What is known about the digital technology currently available and used by women with PCOS for PCOS knowledge, self-management, or social support?” Inclusion criteria were full-length, peer reviewed articles reporting primary research about digital technology used for PCOS-specific knowledge, self- management, or social support by adult (≥18 y) women with PCOS, available in English, and published from the years 2010 to 2025. The year 2010 was chosen to be inclusive of all digital technologies, but with awareness of the rapid nature of technological development in the digital application and website realm [17]. Exclusion criteria were articles that were not primary research (eg, editorials and reviews), research about the pediatric population with PCOS, and studies that used digital technology for intervention recruitment or medical treatment. The pediatric population (≤18 y) was excluded because, although a PCOS diagnosis can be considered in pubescent girls, this age poses diagnostic problems due to characteristics of normal puberty that often overlap with signs and symptoms of PCOS [18]. In addition, the health literacy, design preferences, and access to digital technology of the pediatric population differ from those of adults. We excluded articles about digital technology used for recruitment and by health care providers for diagnosis and medical treatment because we were interested in PCOS-specific digital technology used by women with PCOS for PCOS-specific knowledge, self-management, or social support.

Thus, the participants included women aged 18 years and older with PCOS. The concept for this scoping review was available digital technology used by women with PCOS for knowledge, self-management, or social support. The context is open in terms of geographic regions but specific to digital technology used by women with PCOS and not digital technology used for research recruitment nor in health care settings for diagnosis or procedures.

### Search Strategy

The search strategy was created using Medical Subject Headings terms for PCOS, digital technology, health knowledge, self-management, and social support. Social support was defined as empathy, encouragement, information, and feedback provided by others with shared experiences [19]. Adapting the strategy based on the database PubMed, the databases Embase, CINAHL, and Compendex were also searched for relevant articles. The search strategies used can be found in Multimedia Appendix 1.

### Evidence Screening and Selection Process

The search was completed by 2 reviewers (CB and PJW) using Covidence systematic review software (Veritas Health Innovation). After removing duplicates, the articles were screened by title and abstract. Considering the research question and inclusion and exclusion criteria, copies of the full articles were obtained for studies that appeared relevant. If relevance was unclear from the abstract, the full article was kept for further review. The interrater reliability was strong between the 2 reviewers of the initial screening with a Cohen κ of 0.88. The remaining articles were divided into three-fourth of the research team (CB, PJW, and CH) for individual full-text review. The 3 reviewers collaboratively discussed the inclusion of each article. The selected articles were then read by 2 of the reviewers (PJW and CB) to assign a quality rating. The final selected articles were read again by 3 reviewers (PJW, CB, and CH) to collect data.

### Quality and Risk of Bias Assessment

The JBI, an international research organization and global leader in evidence-based health care, was chosen because it offers checklists and rigorous processes for diverse forms of evidence. Using the JBI checklists, 2 reviewers (CB and PJW) critically appraised each article for methodological quality and risk of bias to increase confidence in the findings. The third reviewer (CH) was available to discuss concerns, of which there were none.

### Data Extraction and Analysis

A data chart was created to compile basic study characteristics (eg, authors, publication year, country of origin, and research design), study purpose, the technology used, outcomes, and conclusions. Furthermore, 3 reviewers (CB, PJW, and CH) extracted data and reviewed the data chart to reduce the chance for errors and bias. The research team met several times to code data into categories and summarize the findings. Findings were viewed through the lens of the Unified Theory of Acceptance and Use of Technology (UTAUT). UTAUT was developed by integrating various behavioral theories to help explain the acceptance and use of digital technology. UTAUT emphasizes four key determinants of intention and usage: (1) performance expectancy (perception that a system helps improve performance or productivity), (2) effort expectancy (perception of ease associated with using the system), (3) social influence (the impact of external social factors such as social norms on use), and (4) facilitating conditions (adequate resources and support to use the system) [20].

### Rigor

In addition to iterative review, study rigor was increased by using a scoping review protocol and the Preferred Reporting Items for Systematic Reviews and Meta-Analyses Extension for Scoping Reviews (PRISMA-ScR) checklist (Checklist 1).

## Results

### Overview

**Figure 1.**
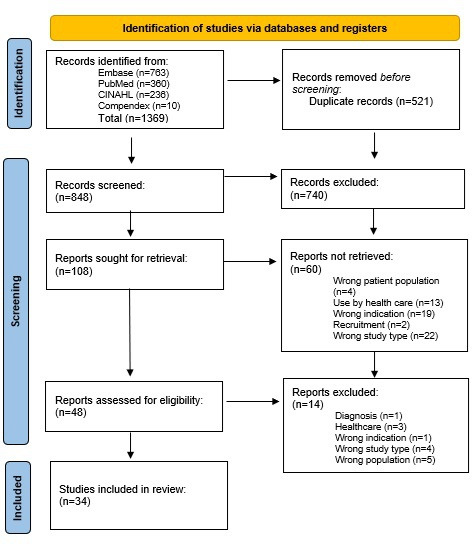
PRISMA (Preferred Reporting Items for Systematic reviews and Meta-Analyses) flow chart.

A scoping review of the literature revealed 34 full-text articles that met inclusion and exclusion criteria (Figure 1).

All selected articles met the criteria on the JBI checklists, and were, therefore, included in the review. Data that were systematically extracted and included in the final summary table were author, publication year, research design, study location, sample size, purpose of the technology, and key findings (Table 1). The studies used various research designs, including cross-sectional and randomized controlled trials. Sample sizes varied widely, ranging from 1 [21] to 416,712 [22]. Given the scope of digital technology, eligible studies were grouped into 7 domains: mobile apps (n=14), internet-based programs (eg, Google; n=6), social media (n=6), SMS text message (n=2), machine learning (n=2), artificial intelligence (AI; eg, ChatGPT [OpenAI]; n=3), and web-based intervention platforms (n=1).

**Table 1.** Characteristics of included studies (N=34).

Author	Year	Design	Location	Sample size, n	Purpose of technology	Findings
Mobile apps						
Alotaibi and Shaman [Bibr R23][23]	2017	RCT^a^	Saudi Arabia	50	To connect women with PCOS^b^ with health care providers and help diagnose PCOS	Increased PCOS awareness
Boyle et al [24]	2018	Cross-sectional	Australia	264	To identify pre-existing mobile apps relating to women’s health or PCOS that may support self-management	16 apps identified: 8 diet only, 3 fertility or menstruation alone or combined with diet, 5 general information
Rodriguez et al [25]	2020	Multiple methods	United States	9	To assess risk factors and diagnose PCOS based on symptoms	Overall, good estimation of risk, but occasional false positives due to overestimation of certain risk factors
Choi et al [Bibr R26][26]	2021	Multiple methods	Korea	30	To promote lifestyle modification and social support for women with PCOS	An integrated app is feasible to develop, but requires further research
Jain et al [22]	2021	Cross-sectional	United States, United Kingdom, India, Philippines, and Australia	416,712	To identify the most prevalent symptoms of PCOS	Common symptoms identified: bloating, high cholesterol, and elevated blood glucose
Sang et al [27]	2022	RCT	China	100	To provide PCOS education, promote lifestyle modification, and provide emotional social support via WeChat (Tencent Holdings Limited)	Statistically greater level of change BMI (87/100, 87.2% lost weight compared with only 68/100, 67.5% in the control) and higher live pregnancy and birth rates
Wang et al [28]	2022	RCT	China	100	To provide theory-based lifestyle modification via an app	Theory-based apps can support weight loss and mental health among women with PCOS
Lee and Lee [29]	2023	RCT	Korea	28	To provide PCOS education and a lifestyle modification program featuring exercise and diet	Apps can support weight loss and reductions in depressive symptoms among overweight women with PCOS
Ou et al [30]	2023	RCT	China	100	To provide health information and patient-provider communication via WeChat and a diet or physical activity tracker via a weight management app	Apps are effective means to provide health information and enable patient-provider communication to help promote health outcomes
Peven et al [31]	2023	Multiple methods	United Kingdom	24	To track symptoms	88% (21/88) exact match between physician and symptom checker classifications, with 100% sensitivity and 75% specificity. Positive predictive value of 80% and negative predictive value of 100%.
Stujenske et al [32]	2023	Cross-sectional	United States	386	To track menstrual cycles and predict fertility	3 main tracking devices: smartphone apps, temperature tracking, and at-home urine hormone tests. 40% (154/386) used for determining fertility, 27% for tracking symptoms (104/386), and 18% (70/386) for receiving reproductive health education
Trépanier et al [33]	2023	Descriptive	Global	119 apps	To provide menstrual and pain tracking	Free mobile apps with varied approaches; overall, good functionality but poor design and lack of evidence-based approaches to pain
Dilimulati et al [34]	2024	RCT	China	80	To provide videos about healthy lifestyle behaviors	WeChat more engaging and produced more healthy lifestyle changes versus metformin alone; however, metformin significantly lowered HOMA-IR^c^
Hamzehgardeshi et al [35]	2024	RCT	Iran	60	To provide health information and counseling via WhatsApp	WhatsApp (Meta) effective means to deliver intervention that promotes healthy lifestyle behaviors
Internet						
Mallappa Saroja and Hanji Chandrashekar [36]	2010	Qualitative	Global	15	To provide information about PCOS-specific symptom management	Quality of information poor; no website with editorial review
Holbrey and Coulson [37]	2013	Qualitative	United Kingdom	50	To provide social support via online support group discussion forums	Positives: emotional, informational social support; negative: source of worry and anxiety
Authier et al [38]	2020	Qualitative	France	211	To provide knowledge and social support to infertile women with PCOS	Positive source of experiential sharing; inaccurate and incomplete health information
Hoyos et al [14]	2020	Cross-sectional	United States	759	To find information about PCOS symptoms, support groups, and other PCOS-specific topics via Google	98.2% (745/759) of women used Google to search their symptoms because >50% women were dissatisfied with care; 18.8% (143/759) sought social support.
Malhotra and Kempegowda [39]	2023	Cross-sectional	Global	—^d^	To increase PCOS awareness and provide PCOS-specific information via Google	Global increase of PCOS awareness especially during PCOS Awareness Month, and increased volume of PCOS searches in recent years
Gomula et al [Bibr R41][40]	2024	Qualitative	United States and United Kingdom	15	To find health information and social support and compare self to “normal” women via Google	Overwhelming amount of information that lacked reliability and cultural sensitivity and created conflict between phenotypes
Social media						
Gour et al [41]	2021	Quasi-experimental	Turkey	41	To provide PCOS-specific knowledge via video-based, structured, educational module posted on preferred social media site	PCOS-specific knowledge significantly increased among those with at least minimum level of health literacy and familiarity with the Internet
Elhariry et al [42]	2022	Qualitative	Global	8	To understand the impact of PCOS influencers on social media platforms	PCOS influencers report motive as PCOS awareness but all with marketing interest
Clarke et al [43]	2023	Cross-sectional	Jamaica	80 YouTube videos	To provide PCOS content on YouTube	About 30% (24/30) of videos created by physicians. Greater global quality score and video power index among videos posted by health care professionals.
Horvath et al [44]	2024	Qualitative	Global	238 videos	To provide PCOS-specific health information via Tik Tok	TikTok attracts considerable engagement; however, most videos provided low-quality information
Naroji et al [45]	2024	Cross-sectional	Global	34,308	To provide PCOS-related information across the social media platforms of TikTok, Instagram, and Redditt	About 1.8million views on top PCOS content on TikTok. Weight and diet most common topics. Interactions with medical providers noted in 30% of content. Reddit most engagement under self-management topics.
Afaq et al [46]	2025	Cross-sectional	Global	12,200 posts	To provide PCOS-specific information and social support via X	Higher-quality content with physicians contributing 30% of the discourse; varied engagement
SMS text messaging						
Jiskoot et al [47]	2020	RCT	Netherlands	183	To promote weight loss among women with PCOS and obesity	Group with SMS text message lost more weight than other groups
Dietz de Loos et al [48]	2021	RCT	Netherlands	183	To provide reminders	SMS text message helpful to remind, encourage, and motivate for lifestyle behaviors
Machine learning						
Zigarelli et al [49]	2022	Cross-sectional	India	541	To predict risk factors and diagnosis of PCOS based on health records for patients and providers	Good prediction accuracy: 80% first run and 82.5% second run.
Karia et al [50]	2023	Correlational	India	—	To track complete menstrual health and predict likelihood of PCOS	72.1%‐90.4% accuracy
Artificial intelligence						
Devranoglu et al [51]	2024	Cross-sectional	Turkey	460 queries	To provide answers about PCOS and fertility treatments via ChatGPT	Although fluctuations in performance, accuracy rated high
Shamanna et al [21]	2025	Case study	India	1	To deliver personalized nutrition by predicting postprandial glucose response	Decreased bodyweight, waist circumference, blood pressure, and fasting insulin after 360 days
Ulug et al [52]	2025	Cross-sectional	Turkey	63 questions	To provide answers to PCOS nutrition-related queries via ChatGPT	ChatGPT provides high quality information; readability difficult for average user
Web-based intervention platform						
Percy et al [53]	2024	Mixed methods	United Kingdom	13 interviews and 58 surveys	To provide theory-based comprehensive PCOS self-management program	Effective for self-management, specifically modest weight loss and reduced depressive symptoms; need for tailoring across lifespan

a RCT: randomized controlled trial.

b PCOS: polycystic ovary syndrome.

c HOMA-IR: Homeostatic Model Assessment of Insulin Resistance.

d Not applicable.

### Mobile Apps

Mobile apps, software applications designed to run on a small mobile device such as a phone, tablet, or wearable, were the most featured digital technology among the selected articles (14/34; 41%). Mobile apps were used for the following reasons: (1) menstrual tracking to assess for the likelihood of PCOS or predict fertility, (2) symptom tracking to assess for risk factors of PCOS, (3) lifestyle modification, (4) PCOS information, (5) social support, and (6) connection with health care professionals. In one article, the authors identified 16 existing PCOS-specific mobile apps that focused on menstrual tracking, nutrition, or general PCOS education [24], while another article identified 119 free mobile apps for menstrual tracking for women with or without PCOS (eg, Flo [Flo Health, Inc] and Clue [BioWink GmbH]) [32]. Findings by researchers in 2 studies about the use of apps featuring lifestyle modification revealed positive outcomes such as decreased waist circumference [28], modest weight loss, and decreased depressive symptoms [29], and positive chemical and clinical pregnancy rates [26] compared with a standard care control group. Apps can be a feasible means to deliver a PCOS-specific lifestyle intervention [29,30,34,35].

### Internet

The category of internet included any type of content electronically transmitted and stored. In 15% of articles (5/34), the researchers studied the Google search browser as used by organizations to promote PCOS awareness [33] and by women with PCOS to search for PCOS health information [30,33,39], nutrition and exercise [14], and social support [14,37,38,40]. In an analytical cross-sectional survey (N=759), researchers reported 98.2% (745/759) of women with PCOS used Google to search their symptoms and 18.8% (143/759) sought support from others who understand PCOS by joining online groups [14]. The Google search engine was reported to be effective at promoting PCOS awareness [39]; however, women with PCOS felt overwhelmed by the amount of information and doubted the information’s reliability [38,40].

### Social Media

Social media refers to interactive online platforms. Researchers who authored 4 of the articles (12%) included information about online platforms: YouTube (Google) [43] and TikTok (ByteDance), Instagram (Meta), Reddit [45], and X, formerly known as Twitter [42,46]. Authors of the article about YouTube described the analysis of PCOS-specific videos (n=80) posted to YouTube. Of these videos, 37% (30/80) were uploaded by women with PCOS and 29% (23/80) were uploaded by health care professionals. Videos posted by women with PCOS had greater popularity as evidenced by total number of likes; however, they had the lowest quality indicator scores compared with those posted by health care professionals [43]. Contrarily, Twitter or X had higher-quality content with physicians contributing most of the discourse [46]. Engagement was higher on TikTok (approximately 1.8 million viewers) than Instagram and Reddit. The most searched topics on TikTok and Instagram were “weight” and “diet” whereas health information and discussion about health care encounters were the most searched topics on Reddit [45]. A conflict of interest among PCOS-specific content on social media platforms was the presence of promotional advertising: 29% (23/80) of all YouTube videos [42], 45% (23/50) of all Tik Tok videos, and 89% (45/50) of all Instagram posts [45]. As such, social media platforms often involve people, called influencers, who affect the purchasing decisions of others because of their authority, knowledge, or position. Elhariry and colleagues [42] identified the top 100 PCOS influencers and interviewed 8 of them. Influencers self-identified as PCOS advocates and strived to post on multiple media platforms. Connections with marketing companies enabled them to spread their message through brand sponsorships.

### SMS Text Messaging

SMS text messaging was featured in 9% of articles (2/34) that reported different analyses from the same study. In the original article, the authors reported the effectiveness of a group-based 3-component lifestyle intervention (nutritional advice, exercise, and cognitive behavioral therapy) to promote weight loss among women with PCOS and obesity. Furthermore, 1 of the 3 study arms received the intervention with an additional 9 months of feedback through SMS text message via their mobile phone [47]. In the second article, they reported on the effectiveness of the intervention on PCOS phenotypical features, such as hyperandrogenism [48]. Overall, the 3-component lifestyle intervention promoted weight loss as compared with the control group, and more so in the group receiving SMS text messages. The authors concluded that SMS text message feedback was useful to remind, encourage, and motivate the women with PCOS to continue the lifestyle behavior and maintain weight loss.

### Machine Learning

In 9% of studies (2/34), apps that included machine learning were developed and tested to track menstrual characteristics [49] and predict the likelihood of a PCOS diagnosis [35]. Machine learning using health care records, patients, and health care providers resulted in 80%‐82.5% accuracy [49], whereas machine learning using various machine learning algorithms ranged from 72.1% to 90.4% [50]. Both study authors noted that currently the accuracy of trackers and PCOS predictors vary widely, especially among free apps, further increasing health disparity in rural areas and among those with lower socioeconomic status.

### Artificial Intelligence

AI is technology that enables computers and machines to simulate human intelligence. Thus, AI systems are made to understand and respond to human language, recognize objects, and learn from new information [54]. Furthermore, 6% (2/34) of articles were about the use of ChatGPT to provide PCOS information; 1 about fertility treatments [51] and 1 about nutrition [52]. In both articles, the accuracy of the information was rated high; however, the readability was a challenge for the average user. Shamanna and colleagues [21] completed a case study examining the use of AI to predict postprandial glycemic response to meals and suggesting alternative meal options to avoid glucose spikes. After 360 days of use, the 38-year-old woman with PCOS decreased body weight, waist circumference, blood pressure, and fasting insulin level [21].

### Web-Based Intervention Platform

Of the 34 articles, the authors of 1 article (4%) discussed the adaptation of a web-based self-management program based on the identified barriers to self-management and psychological well-being reported from women with PCOS [53]. The existing HOPE program was chosen given its research with multiple other patient groups, such as those with diabetes and cancer, the easier accessibility of web-based intervention platform, and the potential to reach many women with PCOS. Using theory and input from end users, 6 modules were created to include health education, self-care, mindfulness, nutrition, communication skills with health care providers, and goal setting. Now called HOPE PCOS, the program offered features to address multiple components of PCOS self-management and was deemed ready for feasibility testing [53]. However, it was not complete as it omitted physical activity or health-specific needs of older women with PCOS.

### Key Findings With a UTAUT Lens

#### Performance Expectancy

The primary reasons women with PCOS used digital technology was to find health information about PCOS treatment and self-management, track menstrual characteristics to determine fertility, and assess the likelihood of a PCOS diagnosis. As expected, women quickly and conveniently found health information with a Google search, yet they reported the volume of information as overwhelming and the content contradictory [40]. Many of the free apps for menstrual tracking lacked reliability [33,50]. Women with PCOS also expected a confidential, secure platform for social support with others who understand PCOS. Overall, women with PCOS, the end users of PCOS-specific digital technology, expected an interactive, comprehensive, evidence-based, and user-friendly platform that assisted with self-management, health behavior tracking, interaction with health care providers, and social support [14,24,26,33,40,53]. Digital technology incorporating these features was found to help promote weight loss and decrease depressive symptoms [28,29,45] and improve biochemical markers such as hyperandrogenism and ovulatory dysfunction [48].

#### Effort Expectancy

Women with PCOS report the expectation of an easily accessible and user-friendly platform [23,25,27,28]. Most apps were found to be easily accessible and user-friendly, although engagement was often low, especially in interventions relying on self-motivation [20,33]. However, non–PCOS-specific apps (eg, Flo and Clue) were criticized for insufficient customization, making the information more difficult to use [22,24]. Only one form of digital technology, the website HOPE PCOS, incorporated end user input during the design phase [53].

#### Social Influence

PCOS awareness was increased through information posted on social media. Some studies noted low involvement in public social support groups [14]. Apps with integrated social functions, such as private networks, demonstrated higher engagement [23,27], especially if the end users shared more in common (eg, life phase) with peers than the illness experience [23,39,40].

#### Facilitating Conditions

Several facilitating conditions were found to be deficient and negatively affected digital technology use among women with PCOS, including lack of accessibility (eg, cost of Wi-Fi or platform) [33,53], content that required high health literacy [46,51,52], and concerns about the reliability of health information [14,24,40,44]. Women with PCOS indicated that the currently available digital technology is helpful but does not address all their needs [24]. Thus, the lack of a comprehensive set of features deterred regular use of any one type of digital technology [24,47,48].

## Discussion

### Principal Findings

Of the selected 34 articles for this scoping review, the researchers’ main goals across the studies included (1) assessing usability, engagement, and performance of different types of digital technology to meet needs of women with PCOS; (2) enhancing knowledge, symptom tracking, and lifestyle modification via digital technology; and (3) predicting the risk of PCOS through machine learning and symptom-tracking apps. A clear expectation of any digital technology was PCOS education. In a large international cross-sectional study of women with PCOS aged 18‐45 years (N=1256), approximately 65% (n=816) reported dissatisfaction with information received from health care professionals and the need for self-directed research [55]. Findings from this scoping review indicate concern about the quality and reliability of online health information. Most online information was posted by other women with PCOS based on anecdotal evidence. Higher-quality content was found on Twitter or X and ChatGPT. The primary users of both platforms have college degrees and represent technical sectors, education, research, or business [56,57]. Unfortunately, as information quality increases, readability for the average user decreased.

A significant amount of PCOS content is associated with commercial interests or promoted by influencers with market interest [40,42,58]. The impact of social media influencers on health outcomes is a growing area of research. In a cross-sectional survey of participants, 59% (137/232) stated that they prefer to follow social medial influencers on social media platforms compared with nonsponsored posts [58]. Most research about influencers have focused on their marketing of diets or the impact of their imagery on body image dissatisfaction [59]. While not entirely negative, this type marketing can be misleading and harmful for individuals with vulnerabilities such as body image concerns [60], which are prevalent among women with PCOS [61].

Consistent with previous studies, most women with PCOS preferred apps for PCOS self-management and personal data tracking given their ease of use in terms of time and space [62]. Many apps are currently available for menstrual tracking; however, most are not equipped to interpret irregular cycles [63], a clinical feature of many women with PCOS [1]. Machine learning advances hold potential to improve the accuracy of menstrual trackers and PCOS predictive models [49,50]. Women with PCOS across the lifespan have also expressed need for reliable PCOS-specific apps that offer an evidence-based, comprehensive approach to self-management, are interactive with both others with PCOS and health care providers, and target multiple outcomes [64,65]. In this scoping review, the app AskPCOS (Monash Centre) and the web-based program HOPE PCOS, were the only digitally based platforms to approximate a comprehensive approach [24,53].

Social media platforms were a common source of social support among women with PCOS because of the asynchronous access to a larger group of individuals who share similar life experiences or interests. Similar to findings from a systematic review about social connectedness among individuals with chronic health conditions [66], engagement diminished over time as women with PCOS sought more common ground than the illness experience, such as life stage (eg, student, mother, and retiree) and, therefore, overall health goal (eg, fertility vs risk reduction of comorbidities) [14,23,26,31,40]. Thus, many social media platforms could benefit from tailoring to reach specific subgroups of women with PCOS, including those with different phenotypes and sociodemographic characteristics (ie, race and residential location).

While digital technology confers several benefits, other facilitating conditions, such as accessibility due to cost or geographic location, remain issues among many women with PCOS. These digital determinants of health encompass technological factors that can influence the access and use of information for self-management, which, in some cases, can potentiate existing sociodemographic inequities in health care [67]. Digital health literacy, or the individual’s ability to find, understand, appraise, and apply health information from electronic sources [68] is an emerging priority as people regularly interface with digital technology for health information. The design and development teams of digital technologies could benefit from consideration of digital determinants of health at the onset and the inclusion of a diverse sample of end users for input on use, useability, and aesthetics. In addition, the development of a system to regularly evaluate, score, and post the reliability of health care information on digital technology could empower women with PCOS as they choose digital technology to use and apply electronic health care information.

Assessing the findings of the scoping review through the lens of UTAUT helps more clearly identify future directions for digital technology used by women with PCOS. For performance expectancy, women with PCOS prefer an interactive PCOS-specific app with comprehensive features, including health care education (eg, diagnosis and treatment), lifestyle modification (eg, physical activity, nutrition, and stress management), personal data tracking, interaction with health care providers, and peer support. They expect reliable, evidence-based information that involves accurate predictions of diagnosis and fertility, promotes positive health outcomes and is unencumbered by commercial interests. For effort expectancy, women with PCOS want an easily accessible, user-friendly, and pleasing interface. For social influence, women with PCOS readily use more popular modes of digital technology to connect with others with PCOS; however, motivation to maintain usage is low due to exclusion of subgroups of women with PCOS and lack of sociodemographic context sensitivity. For facilitating conditions, digital technology with more features and higher reliability may cost more, presenting an economic barrier for some women with PCOS. While mobile phone use is rapidly increasing, including across low- and middle-income earning countries, Wi-Fi access may be unavailable [69].

### Limitations

By using a systematic, comprehensive search strategy, the reach and rigor of the scoping review was increased. However, only records available in English were examined, which may skew the distribution and recognition of studies. The search of only 4 databases was a limitation, as it may have resulted in the omission of relevant literature. However, we included Embase and PubMed, which are considered among the most comprehensive research databases. Digital technology is a rapidly evolving sector, and advancements are made constantly. Additional PCOS digital technologies may have been developed since the time the scoping review was conducted. While wearables, such as fitness trackers, are most likely used by women with PCOS and have potential to aid self-management, no articles were found about wearables specifically for or preferred by women with PCOS.

### Conclusion

The findings of this scoping review revealed that digital health technologies, particularly mobile apps, hold promise for supporting women with PCOS. Future research should focus on advancing user-centered design for apps aimed at managing PCOS, with an emphasis on usability and performance expectancy while accounting for sociodemographic differences and disparities. Health care professionals should also be included to corroborate health care information and increase the likelihood of their endorsement to patients with PCOS. There is potential for digital technology to transform PCOS self-management, but further design and development are needed to optimize these technologies for women with PCOS.

## Supplementary material

10.2196/68469 Multimedia Appendix 1 Search strategies.

10.2196/68469 Checklist 1 PRISMA-ScR (Preferred Reporting Items for Systematic Reviews and Meta-Analyses Extension for Scoping Reviews) checklist.

## Abbreviations

AI: artificial intelligence
JBI: Joanna Briggs Institute
PCOS: polycystic ovary syndrome
PRISMA-ScR: Preferred Reporting Items for Systematic Reviews and Meta-Analyses Extension for Scoping Reviews
UTAUT: Unified Theory of Acceptance and Use of Technology
