# Supplementary material for: Availability and Use of Digital Technology Among Women With Polycystic Ovary Syndrome: Scoping Review
Source: JMIR Infodemiology. 2025 Jun 12;5:e68469. doi: 10.2196/68469 (PMC12178569; doi:10.2196/68469)
Supplement: Multimedia Appendix 1 [file infodemiology-v5-e68469-s001.docx]

# Appendix A

## PubMed Search Strategy

("Polycystic Ovary Syndrome"[MeSH Terms] OR "PCOS"[Title/Abstract] OR "polycystic ovarian"[Title/Abstract] OR "polycystic ovary"[Title/Abstract] OR "poly cystic ovar*"[Title/Abstract] OR "Stein-Leventhal"[Title/Abstract] OR "ovary polycystic disease"[Title/Abstract]) AND ("Artificial Intelligence"[MeSH Terms] OR "Blogging"[MeSH Terms] OR "Cell Phone"[MeSH Terms] OR "Digital Technology"[MeSH Terms] OR "Internet"[MeSH Terms] OR "Mobile Applications"[MeSH Terms] OR "Telemedicine"[MeSH Terms] OR "Wearable Electronic Devices"[MeSH Terms] OR ("Artificial Intelligence"[Title/Abstract] OR "AI"[Title/Abstract] OR "App"[Title/Abstract] OR "Apps"[Title/Abstract] OR "blog*"[Title/Abstract] OR "cell phone*"[Title/Abstract] OR "cellphone*"[Title/Abstract] OR "cellular phone*"[Title/Abstract] OR "chatbot*"[Title/Abstract] OR "chatgpt*"[Title/Abstract] OR "digital electronic*"[Title/Abstract] OR "digital health"[Title/Abstract] OR ("Digital"[Title/Abstract] AND "intervention*"[Title/Abstract]) OR ("Digital"[Title/Abstract] AND "information"[Title/Abstract]) OR "digital platform*"[Title/Abstract] OR "digital technolog*"[Title/Abstract] OR "digital symptom checker*"[Title/Abstract] OR "Ehealth"[Title/Abstract] OR "e health"[Title/Abstract] OR "facebook"[Title/Abstract] OR "influencer*"[Title/Abstract] OR "Internet"[Title/Abstract] OR "Instagram"[Title/Abstract] OR "instant messag*"[Title/Abstract] OR "Linkedin"[Title/Abstract] OR "Mhealth"[Title/Abstract] OR "m health"[Title/Abstract] OR "mobile application*"[Title/Abstract] OR "mobile health"[Title/Abstract] OR "mobile phone*"[Title/Abstract] OR ("Online"[Title/Abstract] AND "forum*"[Title/Abstract]) OR ("on line"[Title/Abstract] AND "forum*"[Title/Abstract]) OR "online health communit*"[Title/Abstract] OR ("Online"[Title/Abstract] AND "information"[Title/Abstract]) OR ("on line"[Title/Abstract] AND "information"[Title/Abstract]) OR ("Online"[Title/Abstract] AND "intervention*"[Title/Abstract]) OR ("on line"[Title/Abstract] AND "intervention*"[Title/Abstract]) OR ("Online"[Title/Abstract] AND "support"[Title/Abstract]) OR ("on line"[Title/Abstract] AND "support"[Title/Abstract]) OR ("Online"[Title/Abstract] AND "self-help"[Title/Abstract]) OR ("on line"[Title/Abstract] AND "self-help"[Title/Abstract]) OR "reddit"[Title/Abstract] OR "short message service"[Title/Abstract] OR "smartphone*"[Title/Abstract] OR "smart phone*"[Title/Abstract] OR "smart watch*"[Title/Abstract] OR "smartwatch*"[Title/Abstract] OR "social media"[Title/Abstract] OR "social network"[Title/Abstract] OR "social networks"[Title/Abstract] OR "social networking"[Title/Abstract] OR "Snapchat"[Title/Abstract] OR "Telemedicine"[Title/Abstract] OR "text messag*"[Title/Abstract] OR "Texting"[Title/Abstract] OR "TikTok"[Title/Abstract] OR "Tumblr"[Title/Abstract] OR "Twitter"[Title/Abstract] OR "Video-based"[Title/Abstract] OR "vimeo"[Title/Abstract] OR "wearable device*"[Title/Abstract] OR "wearable computer*"[Title/Abstract] OR "wearable electronic device*"[Title/Abstract] OR "Web-based"[Title/Abstract] OR "web site*"[Title/Abstract] OR "websites"[Title/Abstract] OR "WeChat"[Title/Abstract] OR "WhatsApp"[Title/Abstract] OR "Weibo"[Title/Abstract] OR "YouTube"[Title/Abstract]))

## Embase Search Strategy

('artificial intelligence'/exp OR 'digital technology'/exp OR 'internet'/exp OR 'internet use'/exp OR 'mobile application'/exp OR 'mobile phone'/exp OR 'social media'/exp OR 'telemedicine'/exp OR 'text messaging'/exp OR 'online support group'/exp OR 'chatgpt'/exp OR 'blogging'/exp OR 'artificial intelligence':ti,ab,kw OR ai:ti,ab,kw OR app:ti,ab,kw OR apps:ti,ab,kw OR blog*:ti,ab,kw OR 'cell phone*':ti,ab,kw OR cellphone*:ti,ab,kw OR 'cellular phone*':ti,ab,kw OR chatbot*:ti,ab,kw OR chatgpt*:ti,ab,kw OR 'digital electronic*':ti,ab,kw OR 'digital health':ti,ab,kw OR (digital:ti,ab,kw AND intervention*:ti,ab,kw) OR (digital:ti,ab,kw AND information:ti,ab,kw) OR 'digital platform*':ti,ab,kw OR 'digital technolog*':ti,ab,kw OR 'digital symptom checker*':ti,ab,kw OR ehealth:ti,ab,kw OR 'e health':ti,ab,kw OR facebook:ti,ab,kw OR influencer*:ti,ab,kw OR internet:ti,ab,kw OR instagram:ti,ab,kw OR 'instant messag*':ti,ab,kw OR linkedin:ti,ab,kw OR mhealth:ti,ab,kw OR 'm health':ti,ab,kw OR 'mobile application*':ti,ab,kw OR 'mobile health':ti,ab,kw OR 'mobile phone*':ti,ab,kw OR (online:ti,ab,kw AND forum*:ti,ab,kw) OR ('on line':ti,ab,kw AND forum*:ti,ab,kw) OR 'online health communit*':ti,ab,kw OR (online:ti,ab,kw AND information:ti,ab,kw) OR ('on line':ti,ab,kw AND information:ti,ab,kw) OR (online:ti,ab,kw AND intervention*:ti,ab,kw) OR ('on line':ti,ab,kw AND intervention*:ti,ab,kw) OR (online:ti,ab,kw AND support:ti,ab,kw) OR ('on line':ti,ab,kw AND support:ti,ab,kw) OR (online:ti,ab,kw AND 'self help':ti,ab,kw) OR ('on line':ti,ab,kw AND 'self help':ti,ab,kw) OR reddit:ti,ab,kw OR 'short message service':ti,ab,kw OR smartphone*:ti,ab,kw OR 'smart phone*':ti,ab,kw OR 'smart watch*':ti,ab,kw OR smartwatch*:ti,ab,kw OR 'social media':ti,ab,kw OR 'social network':ti,ab,kw OR 'social networks':ti,ab,kw OR 'social networking':ti,ab,kw OR snapchat:ti,ab,kw OR telemedicine:ti,ab,kw OR 'text messag*':ti,ab,kw OR texting:ti,ab,kw OR tiktok:ti,ab,kw OR tumblr:ti,ab,kw OR twitter:ti,ab,kw OR 'video based':ti,ab,kw OR vimeo:ti,ab,kw OR 'wearable device*':ti,ab,kw OR 'wearable computer*':ti,ab,kw OR 'wearable electronic device*':ti,ab,kw OR 'web based':ti,ab,kw OR 'web site*':ti,ab,kw OR websites:ti,ab,kw OR wechat:ti,ab,kw OR whatsapp:ti,ab,kw OR weibo:ti,ab,kw OR youtube:ti,ab,kw) AND ('ovary polycystic disease'/exp OR pcos:ti,ab,kw OR 'polycystic ovarian':ti,ab,kw OR 'polycystic ovary':ti,ab,kw OR 'poly cystic ovar*':ti,ab,kw OR 'stein-leventhal':ti,ab,kw OR 'ovary polycystic disease':ti,ab,kw)

## CINAHL Search Strategy

(MH "Polycystic Ovary Syndrome" OR TI (PCOS OR “Polycystic ovarian” OR “Polycystic ovary” OR “poly cystic ovar*” OR “Stein-Leventhal” OR “ovary polycystic disease”) OR AB (PCOS OR “Polycystic ovarian” OR “Polycystic ovary” OR “poly cystic ovar*” OR “Stein-Leventhal” OR “ovary polycystic disease”)) AND ((MH "Artificial Intelligence+" OR MH "Cellular Phone+" OR MH "Digital Technology+" OR MH "Internet+" OR MH "Mobile Applications" OR MH "Telemedicine+" OR MH "Social Media+" OR MH "Blogs" OR MH "Online Social Networking") OR TI (“Artificial intelligence” OR AI OR App OR Apps OR Blog* OR “Cell phone*” OR Cellphone* OR “cellular phone*” OR Chatbot* OR ChatGPT* OR “Digital electronic*” OR “Digital health” OR (digital AND intervention*) OR (digital AND information) OR “Digital platform*” OR “Digital technolog*” OR “Digital symptom checker*” OR Ehealth OR “E health” OR facebook OR Influencer* OR Internet OR Instagram OR “instant messag*” OR Linkedin OR Mhealth OR “M health” OR “Mobile application*” OR “Mobile health” OR “Mobile phone*” OR (Online AND forum*) OR (On-line AND forum*) OR “Online health communit*” OR (online AND “health education”) OR (on-line AND “health education”) OR (Online AND information) OR (on-line AND information) OR (Online AND intervention*) OR (on-line AND intervention*) OR (online AND “peer group*”) OR (on-line AND “peer group*”) OR (online AND self-management) OR (on-line AND self-management) OR (online AND support) OR (on-line AND support) OR (online AND self-help) OR (on-line AND self-help) OR reddit OR “short message service” OR Smartphone* OR “Smart phone*” OR “smart watch*” OR smartwatch* OR “Social Media” OR “Social network” OR “Social networks” OR “Social networking” OR Snapchat OR Telemedicine OR “Text Messag*” OR Texting OR TikTok OR Tumblr OR Twitter OR Video-based OR vimeo OR “Wearable device*” OR “wearable computer*” OR “Wearable electronic device*” OR Web-based OR “Web site*” OR websites OR WeChat OR WhatsApp OR Weibo OR YouTube) OR AB (“Artificial intelligence” OR AI OR App OR Apps OR Blog* OR “Cell phone*” OR Cellphone* OR “cellular phone*” OR Chatbot* OR ChatGPT* OR “Digital electronic*” OR “Digital health” OR (digital AND intervention*) OR (digital AND information) OR “Digital platform*” OR “Digital technolog*” OR “Digital symptom checker*” OR Ehealth OR “E health” OR facebook OR Influencer* OR Internet OR Instagram OR “instant messag*” OR Linkedin OR Mhealth OR “M health” OR “Mobile application*” OR “Mobile health” OR “Mobile phone*” OR (Online AND forum*) OR (On-line AND forum*) OR “Online health communit*” OR (online AND “health education”) OR (on-line AND “health education”) OR (Online AND information) OR (on-line AND information) OR (Online AND intervention*) OR (on-line AND intervention*) OR (online AND “peer group*”) OR (on-line AND “peer group*”) OR (online AND self-management) OR (on-line AND self-management) OR (online AND support) OR (on-line AND support) OR (online AND self-help) OR (on-line AND self-help) OR reddit OR “short message service” OR Smartphone* OR “Smart phone*” OR “smart watch*” OR smartwatch* OR “Social Media” OR “Social network” OR “Social networks” OR “Social networking” OR Snapchat OR Telemedicine OR “Text Messag*” OR Texting OR TikTok OR Tumblr OR Twitter OR Video-based OR vimeo OR “Wearable device*” OR “wearable computer*” OR “Wearable electronic device*” OR Web-based OR “Web site*” OR websites OR WeChat OR WhatsApp OR Weibo OR YouTube))

## Compendex Search Strategy

((('artificial intelligence'/exp OR 'digital technology'/exp OR 'internet'/exp OR 'internet use'/exp OR 'mobile application'/exp OR 'mobile phone'/exp OR 'social media'/exp OR 'telemedicine'/exp OR 'text messaging'/exp OR 'online support group'/exp OR 'chatgpt'/exp OR 'blogging'/exp OR 'artificial intelligence':ti,ab,kw OR ai:ti,ab,kw OR app:ti,ab,kw OR apps:ti,ab,kw OR blog*:ti,ab,kw OR 'cell phone*':ti,ab,kw OR cellphone*:ti,ab,kw OR 'cellular phone*':ti,ab,kw OR chatbot*:ti,ab,kw OR chatgpt*:ti,ab,kw OR 'digital electronic*':ti,ab,kw OR 'digital health':ti,ab,kw OR (digital:ti,ab,kw AND intervention*:ti,ab,kw) OR (digital:ti,ab,kw AND information:ti,ab,kw) OR 'digital platform*':ti,ab,kw OR 'digital technolog*':ti,ab,kw OR 'digital symptom checker*':ti,ab,kw OR ehealth:ti,ab,kw OR 'e health':ti,ab,kw OR facebook:ti,ab,kw OR influencer*:ti,ab,kw OR internet:ti,ab,kw OR instagram:ti,ab,kw OR 'instant messag*':ti,ab,kw OR linkedin:ti,ab,kw OR mhealth:ti,ab,kw OR 'm health':ti,ab,kw OR 'mobile application*':ti,ab,kw OR 'mobile health':ti,ab,kw OR 'mobile phone*':ti,ab,kw OR (online:ti,ab,kw AND forum*:ti,ab,kw) OR ('on line':ti,ab,kw AND forum*:ti,ab,kw) OR 'online health communit*':ti,ab,kw OR (online:ti,ab,kw AND information:ti,ab,kw) OR ('on line':ti,ab,kw AND information:ti,ab,kw) OR (online:ti,ab,kw AND intervention*:ti,ab,kw) OR ('on line':ti,ab,kw AND intervention*:ti,ab,kw) OR (online:ti,ab,kw AND support:ti,ab,kw) OR ('on line':ti,ab,kw AND support:ti,ab,kw) OR (online:ti,ab,kw AND 'self help':ti,ab,kw) OR ('on line':ti,ab,kw AND 'self help':ti,ab,kw) OR reddit:ti,ab,kw OR 'short message service':ti,ab,kw OR smartphone*:ti,ab,kw OR 'smart phone*':ti,ab,kw OR 'smart watch*':ti,ab,kw OR smartwatch*:ti,ab,kw OR 'social media':ti,ab,kw OR 'social network':ti,ab,kw OR 'social networks':ti,ab,kw OR 'social networking':ti,ab,kw OR snapchat:ti,ab,kw OR telemedicine:ti,ab,kw OR 'text messag*':ti,ab,kw OR texting:ti,ab,kw OR tiktok:ti,ab,kw OR tumor:ti,ab,kw OR twitter:ti,ab,kw OR 'video based':ti,ab,kw OR video:ti,ab,kw OR 'wearable device*':ti,ab,kw OR 'wearable computer*':ti,ab,kw OR 'wearable electronic device*':ti,ab,kw OR 'web based':ti,ab,kw OR 'web site*':ti,ab,kw OR websites:ti,ab,kw OR wechat:ti,ab,kw OR whatsapp:ti,ab,kw OR weibo:ti,ab,kw OR youtube:ti,ab,kw) AND ('ovary polycystic disease'/exp OR pcos:ti,ab,kw OR 'polycystic ovarian':ti,ab,kw OR 'polycystic ovary':ti,ab,kw OR 'poly cystic ovar*':ti,ab,kw OR 'stein-leventhal':ti,ab,kw OR 'ovary polycystic disease':ti,ab,kw)) WN ALL)
